# “It’s My Secret”: Fear of Disclosure among Sub-Saharan African Migrant Women Living with HIV/AIDS in Belgium

**DOI:** 10.1371/journal.pone.0119653

**Published:** 2015-03-17

**Authors:** Agnes Ebotabe Arrey, Johan Bilsen, Patrick Lacor, Reginald Deschepper

**Affiliations:** 1 Department of Public Health, Faculty of Medicine and Pharmacy, Vrije Universiteit Brussel, Brussels, Belgium; 2 Department of Internal Medicine and Infectious Diseases-AIDS Reference Center, Universitair Ziekenhuis Brussel, Brussels, Belgium; University of South Carolina, UNITED STATES

## Abstract

Patients with HIV not only have to deal with the challenges of living with an incurable disease but also with the dilemma of whether or not to disclose their status to their partners, families and friends. This study explores the extent to which sub-Saharan African (SSA) migrant women in Belgium disclose their HIV positive status, reasons for disclosure/non-disclosure and how they deal with HIV disclosure. A qualitative study consisting of interviews with twenty-eight SSA women with HIV/AIDS was conducted. Thematic content analysis was employed to identify themes as they emerged. Our study reveals that these women usually only disclose their status to healthcare professionals because of the treatment and care they need. This selective disclosure is mainly due to the taboo of HIV disease in SSA culture. Stigma, notably self-stigma, greatly impedes HIV disclosure. Techniques to systematically incorporate HIV disclosure into post-test counseling and primary care services are highly recommended.

## Introduction

There is still growing concern over the rise in new cases of HIV diagnosed in the European Union (EU), as stated by the European Centre for Disease Prevention and Control (ECDC) [1]. In Belgium there are about 25,879 people living with HIV/AIDS and about 4,550 of the 13,352 patients receiving care are sub-Saharan African (SSA) migrant women. Men having sex with men and SSA migrants are the main groups with a high prevalence of HIV. In 2012, an estimated 1227 new HIV diagnoses were registered in Belgium, of which about 354 diagnoses were in SSA migrant women [2]. It is possible that HIV infection may have occurred outside Belgium, with the positive test results being received in Belgium. According to Belgian law, voluntary testing is the norm but in certain conditions it is very difficult to refuse or avoid testing. This is the case, for example, for asylum seekers. People living with HIV and on combined antiretroviral therapy (cART) are now able to live normal lives after their initial diagnosis. Whereas HIV was very virulent from the early 1980s onwards, the use of combined antiretroviral therapy (cART) has now turned HIV into a chronic but treatable disease [3].

Generally people with chronic illnesses like diabetes, mental illness, asthma and hypertension have to learn to incorporate the management of their illnesses into their daily lives. As patients of chronic diseases comprehend their diagnosis, they are at the same time confronted with issues of how to integrate their illnesses into their daily lives and create new identities for themselves. A striking difference between HIV and other chronic diseases is that HIV is highly infectious and predominantly transmitted through sexual contact [4]. HIV/AIDS is still not yet socially accepted as a ‘normal’ chronic disease, making HIV/AIDS patients vulnerable, stigmatized and disinclined to disclose their disease.

Stigma has been defined as “the phenomenon whereby an individual with an attribute which is deeply discredited by his or her society is rejected as a result of the attribute” [5]. Illness-related stigma and discrimination, along with their public health implications, have an impact on a globalized environment [6–8]. The concept of stigma had been studied in relation to illnesses before the emergence of HIV/AIDS [9]. Stigma has been shown to act as a barrier to prevention, treatment and care [10] because of the feelings of shame, blame, guilt and social isolation [11]. Stigmatizing attitudes, whether experienced [12], perceived [13] or internalized [14] are often important issues of concern for individuals with chronic and incurable health conditions like the HIV/AIDS disease [15–20], and can have adverse effects, including difficulties in personal relationships, disruption of vocational, professional and educational goals and, crucially, delay in seeking help [21,22].

Negotiating when and how to disclose chronic and life-threatening diseases is never easy, neither for treating physicians nor for diagnosed patients [23]. In the case of HIV, the disclosure of a positive diagnosis is, even more than in other chronic diseases, problematic, because of its discredited and discrediting aspect [5,24]. It is thus extremely difficult and challenging to live with a life-changing event of this kind in some cultural settings,[25] especially as the possible revelation of one’s HIV positive status creates an internal struggle about whether or not to disclose [26,27].

Legally, there is no universal requirement to disclose HIV. Countries decide on how and when HIV disclosure is deemed obligatory. The dual requirement of the *‘duty to warn’* of HIV positive status to prevent health risks to a third person and the ‘*duty of confidentiality*’ exists in some states in the United States of America,[28] with unclear boundaries [29]. In Canada HIV- positive status disclosure is obligatory before engaging in any sexual activity with the possibility of HIV transmission [30]. European Union countries have no legal obligation to disclose HIV positive status. Protecting private data is a more fundamental right here [31]. In Belgium, likewise, the law clearly protects individuals’ right to privacy, which gives people living with HIV/AIDS the right to choose when and how they want to disclose their HIV positive status. This law is applicable to Belgian nationals as well as immigrants [32].

Migration entails movement of people and it is often pushed by the need to explore new opportunities, escape from hunger, armed conflicts or violence [33]. Movement from one place to another tends to involve some degree of trauma and migrant populations react differently to constitutions, health systems, cultures and languages other than that of their places of origin [34]. Most emigrants from low-resource regions such as sub-Saharan Africa (SSA) leave their home surroundings not only with socio-economic problems but also with health care problems that necessitate urgent health care solutions in their host countries. For example, a majority of migrants in the EU diagnosed HIV positive come from countries with generalized epidemics and were infected in their countries of origin. The vulnerability of many migrants with HIV/AIDS is exacerbated by the socio-cultural, legal, and policy differences, as well as unfamiliar languages, in their host countries [35].

Women are more vulnerable than men when diagnosed HIV positive. Women are the group most often infected or affected by HIV/AIDS and their gender, sexuality and often lower socio-economic status makes them more vulnerable than men. Moreover, HIV-positive women are in general more afraid of disclosing their status than men, because they are more concerned about their reproductive abilities and roles as caregivers, and they fear far-reaching consequences of disclosure [36]. SSA migrant women living with HIV/AIDS in Belgium and in other EU member states share these common concerns and fears about disclosure of HIV status. Moreover, they are often poor, come from countries with a high prevalence of HIV/AIDS and, above all, are migrants with social, economic and legal issues to deal with in their recipient countries.

Cultural background and gender may influence HIV/AIDS disclosure decisions. Previous studies have shown that voluntary or involuntary disclosure entails social risks including family and community rupture [37]. Decisions to disclose may depend also on agency [38,39], that is, the individual’s ability to act as s/he chooses and refuse multiple relations or insist on condom usage without fear of reprisals (choice-enabled). Likewise, some people are unable to make choices (choice-disabled) because of their socio-economic or educational level. Choice-disability is lack of freedom of choice for those who, for example are victims of intimate partner violence or destitution [40,41]. With regards to HIV/AIDS, choice-disability is a blind spot in HIV/AIDS prevention and is probably a pivot of the epidemic among sub-Saharan African women who bear the brunt of the disease.

Disclosure of one’s HIV status can is considered an important health-promoting support strategy that can avert further HIV transmission [42]. Failing to disclose HIV positive status to sexual partners may be significantly risky [43] and it is known that HIV status disclosure is vital for the enhancement of treatment adherence. Several studies have depicted the importance of HIV disclosure in preventing HIV transmission [44,45] but knowledge about disclosing or not disclosing HIV positive status, especially among SSA migrant women in Belgium, is lacking.

Other studies on the attitudes and behavior of SSA migrant women in relation to HIV disclosure suggest that disclosure decisions are influenced more by the women’s socio-cultural background and less by their new environment [46–49]. This study is important considering the fact that about one third of HIV/AIDS patients receiving care in Belgium are SSA migrant women. The objective of this study is to explore the extent to which SSA migrant women disclose their HIV positive status, the reasons for disclosing or not disclosing and how they deal with disclosure or non-disclosure.

## Methods

### 2.1 Study design

A qualitative study based on semi-structured interviews with SSA migrant women receiving HIV/AIDS treatment and care in Belgium, either identified by health care professionals from consultation lists or self-identified while attending HIV conferences as people living with HIV/AIDS. Follow-up interviews were conducted four months after the first interviews. In addition, their treating professionals were interviewed, observations were made during consultations and information from the hospital records as to their age and year of diagnosis to complement data was obtained. Where women refused to be interviewed, the healthcare providers systematically asked the patients their reasons for refusal. These reasons were communicated to the researcher who took note of the patients' reasons.

### 2.2 Participants

The recruitment of participants was done in two phases. Firstly, recruitment was done through healthcare professionals treating the women at an AIDS Reference Centre (ARC) at a university teaching hospital. The second phase of recruitment involved snowball sampling of SSA migrant women self-identified as living with HIV/AIDS during conferences on HIV/AIDS in Belgium. During the coffee break, the researcher (AEA) talked to a woman who was willing to be interviewed. She was also asked to introduce the researcher to other participants at the conference.

All the women invited were adults, aged 18 years and above, speaking French or English and receiving treatment in Belgium. Only women originating from SSA who had been diagnosed with HIV/AIDS were included in the study. Participants received no financial incentive. Patients only recently diagnosed, within a period of less than three months, were excluded because of the great emotional impact of finding out one is HIV positive.

### 2.3 Data collection and Study procedure

Data collection for the study was done between April 2013 and December 2013. Health care professionals identified patients that met the inclusion criteria from the consultation list, informed them about the study and invited them to participate. The treating physicians briefly explained the aim of the study to patients. Participants recruited from HIV conferences were approached and invited by the researcher to participate in the study. In both cases where they agreed to participate, they signed the informed consent forms. Interview questions were focused on whether or not disclosure was made, reasons for disclosing or not and how they coped with status disclosure or non-disclosure, allowing the participants to guide the conversation to a great extent. During the interviews with the patients, one main question asked after the background information questions was: “After the doctor revealed to you that you are HIV positive, did you disclose your positive status to anyone? If yes, why, to whom and when did you disclose; if no, why not?” The interviewer asked probing questions following the trend of the conversation.

All but one interview was recorded digitally. All interviews were conducted in French or English. As regards observations, the treating physician asked patients if the researcher could be present for observations during consultations and notes were taken based on what was observed. For example, conversations about new illness symptoms, uses of medications, patients’ opinions on current treatment and side effects were noted by the observing researcher. Additionally, certain medical examinations such as blood pressure and weight were also noted. During the data collection phase, feedback from health care professionals as to participants’ reasons for accepting or refusing to be interviewed was also noted and included in the analysis process.

### 2.4 Data analysis

All interviews were transcribed verbatim in French or English. The transcriptions and field notes from observations were then reviewed and coded in preparation for thematic content analysis. Open coding was used to retrieve themes in line with the study objective and, based on grounded theory, an inductive process was used to identify themes as they emerge from the data. This is also known as the “bottom-up approach” [50,51]. Themes related to the topic were identified by constant comparison until saturation was reached. Two researchers (AEA and RD) read and analyzed several interviews and then compared and discussed their findings until there was consensus about the codes and their meaning [52]. In this study, the use of thematic analysis was important in the identification of new themes that recurred in the data and that could eventually produce a bigger picture leading to universal observations [53]. Quotations from the data were presented with any potential identifiers removed.

### 2.5 Ethical statements

The study was approved by the Ethics Committee (EC) of the Universitair Ziekenhuis Brussel, Belgium (Approval number B.U.N. 143201215911) and the Institutional Review Board (IRB) of the Institute of Tropical Medicine, Antwerp, Belgium (Approval number IRB/AB/ac/141). The informed consent form, written in plain language, was approved by the EC and IRB, and was used for documenting the participants’ consent. The approved informed consent form made provisions for participants to include their names, contact addresses and signatures if they agreed to participate. Following the confidentiality section on the approved informed consent form, participants could opt not to sign or give contact addresses. The rationale and data collection methods of the study were explicitly stated on the informed consent form that was presented to each participant, who could ask for more explanations when necessary. Participants provided written and signed informed consent. Participants who could not read and understand the terms on the consent form asked the interviewer to read the procedure of the consent to them before signing the informed consent form. Patients’ anonymity was assured and they could withdraw from the study at any time. Refusal to participate by no means had any influence on the standard care that the patients were receiving. Interviews were conducted at a place of the participants’ choice. The ARC that provided space for recruitment and data collection played no role in the study design, data analysis or in the preparation of the manuscript.

## Results

### 3.1 Characteristics of participants

In total 28 women from 14 countries in SSA with diverse socio-economic and educational background were interviewed and five observations were conducted during consultations. Four treating physicians and an HIV/AIDS therapist nurse of these patients were interviewed individually on their perspectives on treating SSA women with HIV/AIDS. Interviews lasted between 30 minutes and 1 hour with the patients and a one-off interview of between 15 minutes and 1 hour was held with the physicians.

Seventeen of the SSA women had learnt of their HIV-positive status during routine health check-ups at prenatal visits, when considering having children or in connection with other health issues during their residency, legal or not, in Belgium. Seven other women knew of their HIV positive status while in their home countries. Seeking effective treatment and care was the main reason for migrating for these seven women. Four women believed they contracted HIV in Belgium, considering the fact that they were either born or became sexually active in Belgium (Table 1). Of the twenty-eight women interviewed, sixteen had had a higher or university education, ten had had secondary education, one had had primary education and one had had no formal education. All spoke and understood French or English, so there was no need for a translator. Twenty-two of the 28 participants had “intimate partners” (IP) who were husbands (n = 15), live-in boyfriends (n = 1) or casual sexual partners (n = 6). Six participants had no intimate partners and were living alone with their children after having been widowed, abandoned by a spouse or making personal decision for complete abstinence as a result of their HIV-positive status.

**Table 1:** Characteristics of patients (*n = 28*).

Patients	Family status	Child	Education	Employment	Reported mode of transmission	Interview setting	Place of diagnosis of HIV+
1	Couple/live-boyfriend	No	Secondary	Student	Heterosexual	Clinic	Africa
2	Couple/husband	Yes	University	Jobseeker	Heterosexual	Home	Belgium
3	Single/IP/casual sex partners	Yes	Secondary	Disability	Heterosexual	Home	Belgium
4	Single/no IP	Yes	Secondary	Jobseeker	Heterosexual	Clinic	Belgium
5	Couple/husband	Yes	Secondary	Yes	Heterosexual	Clinic	Africa
6	Couple/husband	Yes	High School	Yes	Heterosexual	Clinic	Belgium
7	Single/no IP	Yes	No formal education	Retired	Heterosexual	Clinic	Africa
8	Couple/husband	Yes	Primary	Yes	Heterosexual	Clinic	Belgium
9	Couple/husband	Yes	University	Yes	Heterosexual	Clinic	Belgium
10	Single/IP/casual sex partners	No	High School	Yes	Heterosexual	Clinic	Belgium
11	Single/no IP	Yes	University	Yes	Heterosexual	Clinic	Belgium
12	Single/IP/casual sex partners	Yes	University	Yes	Heterosexual	Home	Infected in Belgium
13	Couple/husband	Yes	University	Yes	Heterosexual	Home	Belgium
14	Couple/husband	Yes	High School	Yes	Heterosexual	Home	Belgium
15	Couple/husband	Yes	Secondary	Yes	Heterosexual	Clinic	Belgium
16	Couple/husband	Yes	Secondary	Yes	Heterosexual	Clinic	Belgium
17	Couple/husband	Yes	High School	Yes	Heterosexual	In my car	Belgium
18	Single/IP/casual sex partners	Yes	Secondary	Yes	Heterosexual	Home	Belgium
19	Couple/husband	Yes	University	Yes	Heterosexual	Home	Belgium
20	Single/IP/casual sex partners	No	University	Jobseeker	Heterosexual	Home	Africa
21	Single/no IP	Yes	High School	Retired	Heterosexual	Park	Africa
22	Couple/husband	Yes	University	Disability	Heterosexual	Hospitalized	Belgium
23	Single/IP/casual sex partners	No	Secondary	Jobseeker	Heterosexual	Coffee shop	Infected in Belgium
24	Single/no IP	Yes	Secondary	Retired	Heterosexual	Home	Africa
25	Couple/husband	Yes	High School	Retired	Work setting	Clinic	Belgium
26	Single/no IP	No	University	Jobseeker	Heterosexual	Clinic	Infected in Belgium
27	Couple/husband	Yes	University	Disability	Heterosexual	Clinic	Africa
28	Couple/husband	Yes	Secondary	Jobseeker	Heterosexual	Clinic	Belgium

Thirteen participants had HIV-negative intimate partners, 10 intimate partners were HIV positive and the HIV status of five partners was unavailable at the time of the interviews. Seven of the 28 participants were less than 40 years old. The youngest participant was aged 23 and the oldest was 67. Two participants provided written consent but did not sign the informed consent form because they believed that it was unnecessary and their identities would be disclosed. The reported mode of transmission of the HIV infection was heterosexual for twenty-seven participants who were interviewed; only one participant reported work-related transmission while working as a nurse in a refugee camp following armed conflict. The preferred venue for interviews was the clinic where most interviews were conducted. Eight interviews were conducted at the homes of study participants and one in a coffee shop located at a railway station and two interviews were conducted in a park and in a car. One participant was hospitalized at the time of interview.

Many participants reported the importance of secrecy that is, revealing their HIV positive status only to a “selected few” if possible; and hiding anything like medications that might identify them as HIV/AIDS patients (concealment). We structured our findings in the following way: characteristics of the participants and their choice to disclose or not, divided into the following subcategories—reasons to disclose, reasons not to disclose, coping strategies and experiences of disclosure.

### 3.2 To disclose or not to disclose

**Fig 1:**
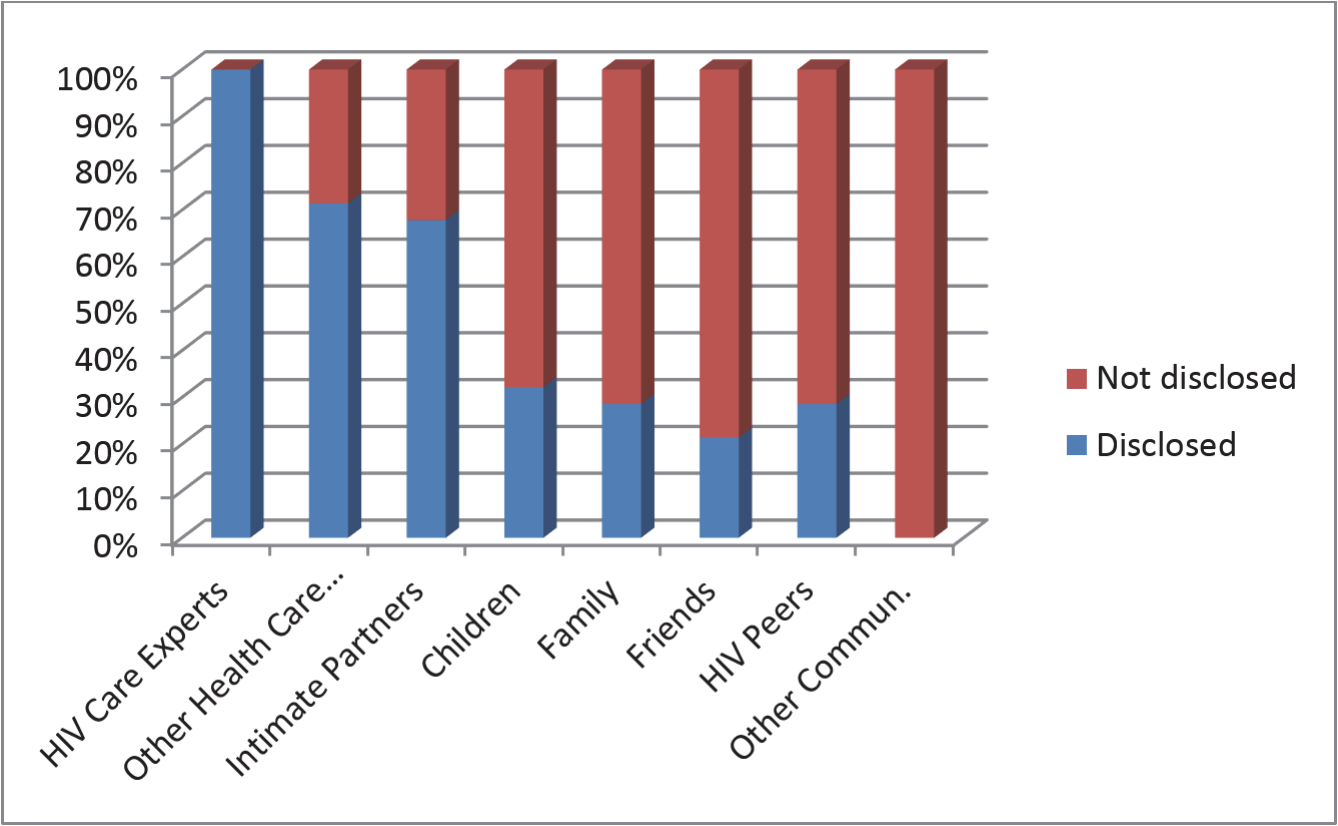
Proportion of patients (as shown in Table 2) that disclosed HIV/AIDS status and to whom they disclosed.

A prevalent theme in the data was disclosure and the women reported that they were confronted with the problem of who to disclose their HIV status to, how and why. The women differed in the way they disclosed their HIV positive status after being diagnosed (Table 2 and Fig. 1). All participants reported selective disclosure to general practitioners (GPs), dentists, pharmacists, intimate partners (husbands, live-in boyfriends, and [casual] boyfriends who lived apart from them), children, selected family members and friends (relevant others). Most participants reported receiving support and empathy from intimate partners, families and friends after disclosure and believed that this contributed to their general well-being and life with HIV/AIDS. Eight participants reported hiding their HIV/AIDS positive status from everyone except healthcare providers directly involved in the treatment and care of their HIV/AIDS.

**Table 2:** Selective disclosure *(n = 28)*.

HIV status	HIV care experts	Other Health care professionals	Intimate Partners	Children	Family	Friends	HIV Peers	Other community
**Disclosed to**	28	20	19	9	8	6	8	0
**Not disclosed**	0	8	9	19	20	22	20	28

Participants associated choice-ability or disability with their disclosure decisions. The women chose to whom they wanted to disclose to, depending on the trust they had in the person. The gender of the person was not a main issue in their decision to disclose. What was paramount was whether that person could keep “the secret” or not.

### 3.3 Reasons for disclosure

Participants reported different reasons for disclosing their positive status, especially to their intimate partners. Some very important themes that recurred were the desire to have children, the need to continue their motherhood and caregiving roles; informing people to avoid transmitting the disease and the need to talk.

#### The desire to have children

Motivations related to fertility and caregiving were the same for the participants. Participants who wanted children and those who were already mothers discussed how important socially and culturally it was to continue in their roles as mothers and caregivers despite living with HIV/AIDS. The women’s resilience was evident in a treating physician’s recounting of his experiences with SSA HIV/AIDS women:

Disclosing to an intimate partner is a big issue in African communities. The women ask questions like ‘How will I live with this disease or can I still live a normal life’? It is easier to convince women to start treatment because they want to have children in the future and that is the reason they are more ready and less inhibited than SSA men to start treatment. If they are pregnant the decision is quickly made. They don’t only have to treat themselves but they have to protect their unborn babies. To the women I think that it is more important to protect their babies than to protect themselves. (Treating physician 2)

#### Informing people to avoid transmitting the disease

The participants who disclosed also said there was the need to inform to prevent transmission. Those who disclosed to their cohabitants or casual sex partners affirmed that it was not an easy decision to make but that it was imperative for them to disclose to their partners as a sign of honesty in the relationship. The interviewees indicated that they informed their intimate partners of their HIV-positive status because they wanted to prevent HIV transmission to their partners and to be able to manage the disease with the support of their partners. Condom usage, fidelity and treatment adherence could be freely discussed if partners were aware of their status. One of the participants commented:

I disclose to protect my partner from HIV infection. But the problem is that when you have this disease and you tell a partner in order to decide how to manage and to prevent the transmission of the disease, the person disappears. This makes me not want to tell another person I am interested in having a relationship with. (Participant 28, first interview)

#### The need to talk

Agreeing to participate in the study gave some of them the opportunity to talk confidentially about their lives with HIV/AIDS. The participants reported the experience of feeling better after disclosure. Many participants reported having had the need to disclose to someone who was not a medical professional, but that had been difficult. This is what a participant said:

I have never spoken about my illness to anyone besides health care providers or in the support group. I feel better now (Participant 20, first interview)

One explained: “I am happy that you could listen to me without judging or criticizing me”. (Participant 23, first interview)

Another woman had this to say when asked why she agreed to participate in this study:

HIV remains a taboo subject and I hope this study may help increase the awareness of the seriousness of the disease, especially among the African communities in this era of highly active antiretroviral therapy. There is still no cure. No one is safe. It is my effort to ducate and motivate changes of behavior in the wider public. (Participant 17, first interview)

### 3.4 Reasons for keeping HIV-positive status secret: non-disclosure

The main reasons why participants want to keep their HIV positive diagnosis secret are: fear of stigma and discrimination, shame, fear of disrupting relationships, rejection, violence and abandonment. Concerns about confidentiality and distrust of other SSA migrants living in Belgium as well as their compatriots in Africa was also evoked as fuelling the determination to hide their HIV status.

#### Fear of stigma and discrimination

Usually participants only disclosed to health care professionals at ARCs because they needed their treatment and care. The embedded fear of being stigmatized was at the center of all the discourses. Most of the women feared being mocked after their HIV positive status was revealed to them. They anticipated stigmatized reactions from people who were still unaware of their HIV-positive status, as one woman explained:

I don’t understand why people who are unaware of their HIV status or who are not sick should mock something as serious as HIV. If they know that you are HIV positive, you are pigeon-holed. That is why we don’t want people to know. Those who know of your HIV positive status will exclude you from their lives. It is terrible. (Participant 25, first interview)

Another participant had this to say in relation to positive HIV diagnosis and self-image:

All that I fear are the side effects of the medications and the visible signs of the disease on my body because people are very inquisitive, especially among us Africans. People will look at you and say ‘you see she is always sick and has boils on her body, what is happening to her’? Are you sure she does not have HIV/AIDS? (Participant 3, first interview)

#### Shame

To all the women, shame was one of the major main reasons why they did not like to disclose their status. One said:

It is shame because HIV is contracted through sex and sex is a taboo for some Africans. There is no other reason. If I tell him/her, she/he will spread it everywhere. We have not yet reached that stage of removing the shame of being HIV infected. It is shame and shame kills. (Participant 2, first interview)

A health expert made a similar allusion to shame as an important issue in the decision not to disclose:

I know that in the beginning when we had medications that had to be kept in the refrigerator, it was a big issue for African migrant women because they complained that when their friends come to their homes, they freely opened the refrigerator. They could have found their medications which would have revealed that they had HIV/AIDS (HIV therapy counsellor).

#### Fear of disrupting relationships, abandonment and violence

Some women feared that disclosing their HIV/AIDS status would disrupt family roles and routines, as some women experienced abandonment and emotional violence from uninfected spouses after status was disclosed. More than half of the participants revealed that they accepted the risky sexual behavior of their intimate partners for financial, social and emotional reasons. Gender-based inequality was verbalized by a woman:

After testing positive, my husband confessed to his infidelities and asked for forgiveness. I stayed with him because my child was very young. It is deplorable that men don’t take precautions to protect themselves, their wives and eventually their unborn babies from contracting HIV. They don’t hesitate to transmit to other women. It is a vicious circle. (Participant 21, first interview)

To continue their mothering and partner roles, some participants deliberately concealed their status from their partners and children. Some women reported having told their children that it [HIV/AIDS] was cancer or another socially acceptable disease to avoid rejection or harassment and other forms of stigma and discrimination. When asked why children are not aware of their mother’s HIV status, one woman said:

My children are not aware of my HIV positivity. I told them that I have cancer and pulmonary tuberculosis and I would like to keep it that way. More so, I don’t want to worry my children. Once my son asked what I was suffering from and I told him that I had a problem in my head and heart because of the potassium treatment I was taking. I know my children. They are very sensitive. (Participant 3, follow-up interview)

They live trying to handle treatment adherence and recurrent opportunist infections while maintaining relationships and stress from repercussions of status disclosure. To emphasis the need for secrecy, one participant expressly preferred to be interviewed at the clinic immediately after her consultation because she could not talk freely at her home without her children learning of her status. This is what she had to say when asked why she had not disclosed her status to her adult children:

I live in hiding from people and I want to protect my children from the worries of this illness (HIV). They will be very worried if they know my status because they are very fragile and emotional. When they see me sick with simple cold, they think ok, I will get well, it’s just a cold. Keeping my illness secret is just to protect them. That’s what I say to myself. Later, I will tell them but not now. (Participant 4, first interview)

This participant found no benefits in her children knowing her status. She perceived the emotional reaction to be more significant than the benefits of disclosure.

#### Breach of confidentiality and distrust

Lack of confidence and trust among the African diaspora was also evoked by all the participants. They were concerned with the fact that their HIV positive status would become public both in Belgium and in Africa. Distrust for the African communities in Belgium was often given as a reason why invitations to participate in the study were rejected by some patients. Coming from a region with a generalized epidemic, it was certain that the participants themselves knew someone who was living with or had died from HIV/AIDS either in their countries of origin or Belgium. They were all susceptible to gossips in their families and socio-cultural and religious factors, as one reported:

It is very difficult to listen to them talk about HIV/AIDS because when you listen to some of them, HIV is just something to be mocked at. They give the impression that there is no one in their circle with HIV when they speak, but we often hear that one of their relatives died of AIDS. It is mockery, mockery, mockery. When you have HIV and you’re in the presence of such people, you are not at ease. We are forced to shut up and not discuss HIV/AIDS. (Participant 5, first interview)

All participants reported having witnessed negative attitudes and behaviors towards other people with HIV infection that enhanced their determination not to disclose. This is what one participant said:

While in the asylum center, I saw and heard how people whose HIV status was known were treated and I swore that no one in that center would know that I was HIV positive. (Participant 1, first interview)

### 3.5 Coping strategies

Since hiding their status was for pivotal all participants, they developed specific coping strategies to keep their status hidden. Secrecy, concealment, social isolation and distancing emerged as important themes.

#### Secrecy

Participants described how they hid their HIV status from those they did not want to disclose to, especially intimate partners who did not live with them and children. Sixteen participants were interviewed at the clinic because they felt comfortable in this setting. In keeping their illness secret, they felt stronger and believed they could better manage their illness. They had only the burden of keeping their secret. However, disclosure to husbands and live-in intimate partners was implicit and evident in nineteen of the twenty-eight participants’ discourses. Most of them took their medications in the presence of their intimate partners who were part of their HIV trajectory. The intimate partners, with knowledge of their partners’ status became “keepers of the secret” [54] as illustrated by the spouse of a participant who encouraged her to be interviewed without signing the informed consent form.

Children were not the only ‘relevant others’ who did not know of “the secret”. Conversely, three participants who were not cohabiting with their intimate partners did not see it necessary to disclose their HIV status. An illustration of this attitude is evidenced by what a participant said when asked if she had disclosed her status to her partner:

My partner is not aware of my HIV positive status. I want to inform him but I think it is not necessary because my viral load is undetectable and I am no longer infectious. My partner was tested negative. So I cannot tell him that I am HIV positive. (Participant 9, first interview)

Nine participants reported that their concerns for the emotional stability of their ‘relevant others’ (especially adult children) deterred them from revealing their positive HIV status and that they were reluctant to discuss health problems for fear of worrying or becoming a burden to their family members. They wanted disclosure to be timely, but when and how was the puzzle they still had to solve.

#### Concealment from “relevant others” and outsiders

Some participants made it clear that concealment was a strategy they used in coping with the HIV disease and treatment. One participant who has been living with HIV/AIDS for more than 20 years reportedly concealed all her daily doses of ART in a plastic vial which she hides behind other bottles and containers in her refrigerator. In the course of our discussion, she brought out the little white vial and said:

People [family, friends, children and intimate partner] are not stupid. I put all my tablets in this box and I know by heart which one to take at any time of the day. I remove the medications from their original packages and put them in the plastic vial and hide them behind mayonnaise bottles. In this way no one knows what medications I take. That is why I hide my medications especially from my daughters. My children don’t know that I have HIV/AIDS. (Participant 3, first interview)

#### Social isolation and distancing

Hiding their disease from outsiders was better handled by distancing. They attended social, cultural and religious gatherings and interacted with people when they wanted, as long as there were no visible signs of AIDS. A participant said:

I go to church but I have not been able to tell anyone that I have HIV. When I say I have a headache or am not feeling well, my friends tell me I should go to the hospital and do the HIV tests but I say nothing to them. I live in hiding. If you tell your friends that you are HIV positive, you will be humiliated and looked at as if you have sinned. We prefer to talk to a doctor. (Participant 4, follow-up interview)

Participants also discussed the fact that they isolated and distanced themselves from networking with people who did not know of their HIV positive status. A participant described it this way:

It is not people who distanced themselves from me because they did not know that I am HIV positive. I distanced myself from people. I don’t want to mix with people because there is something in me called HIV. I fear it can be read on my face. (Participant 1, follow-up interview)

### 3.6 Experiences of Disclosure

Disclosure was not without consequences. The participants reported experiencing negative or positive consequences as a result of disclosure depending on what relationship they had or the partner’s HIV status at the time of disclosure. Those who were together before the diagnosis usually had more positive experiences. Positive consequences reported were HIV information-seeking behavior, support and empathy but on the other hand, rejection, abandonment, and violence were the negative consequences of the revelation of positive status.

#### Positive experiences of disclosure

As a result of openness of diagnosis, love and non-judgmental attitudes were experienced from those with whom participants shared their HIV positive diagnoses. Participants also discussed their eagerness to learn more about HIV prevention, treatment and care from their HIV experts.

#### Support and empathy

All participants that disclosed reported that the support and empathy they received from intimate partners, families and friends after disclosure of their positive status contributed to their general well-being and life with HIV/AIDS. In discussing support and empathy, a participant said:

“I disclosed to my partner before marriage and he said, with available treatment and preventive measures we could manage the illness. I am living well with my illness with the support I get from my partner”. (Participant 2, first interview)

It was much easier to take one’s medications and adhere to treatment after disclosure as acknowledged by one participant:

My husband always reminds me to take my pills whenever I forget to take them. He encourages me to go for my hospital appointments by accompanying me to every visit. His support helps me to follow my treatment strictly and remain healthy. (Participant 13, first interview)

#### HIV information-seeking behavior

Participants talked about seeking information from their HIV treating personnel. The women in the study appeared to be fairly well oriented about the nature of their disease and treatment trajectory, as supported by a treating physician’s words:

The patients come with so many questions. I have to explain what having and living with HIV/AIDS entails. I don’t have the opportunity to get to know the patients better because they want to know a lot from us about HIV. (Treating physician number 4)

#### Negative experiences of disclosure

The negative consequences of disclosure that were reported by a majority of the women were: stigma and discrimination, disrupting relationships, rejection, violence, abandonment and gossips in health care settings, family and the community.

#### Stigma and discrimination in healthcare settings

In addition to the negative experiences from the community and families, participants discussed what they considered discriminatory in healthcare settings. Ten women reported experiencing stigma and discrimination from doctors and nurses not directly involved in their HIV treatment and care. They did not specify the origin of the healthcare providers. In relation to the experiences of stigma and discrimination in healthcare providers, one said:

I was always the last to leave the hospital service even if my appointment was in the morning. I was never told the truth. People who were not HIV positive were treated first and I was always the last. I used to cry a lot and quarrel with the nurses who told me that my case was special. I never liked going for consultations because I did not know when I would be seen by the treating physician. A doctor told me that because of my HIV/AIDS the materials had to be sterilized after use and he made me understand why I was always the last person to be consulted. I found it discriminatory. (Participant 22, first interview)

Another comment from a participant to support stigma and discrimination in health settings:

I was refused the opportunity to become pregnant ‘in vitro’ in a fertility clinic because I am HIV positive. I was very disappointed because I wanted to become a mother. I left that clinic and went to another clinic that agreed to make my dream of becoming a mother come true. (Participant 5, first interview)

#### Violence from intimate partners

Eight women said that they experienced physical and verbal violence as discussed by one woman:

My divorce was very difficult and publicized. Someone came and told me that my ex-husband used to beat me because of my HIV. Yes, he battered me. I supported [= endured] a lot of marital violence. We had a lot of problems. (Participant 10, first interview)

Another participant had this to say on HIV-related violence:

My former partner [husband] told everyone who knew us that I have AIDS and threatened to ask for custody of our children whenever there was a dispute between us. He verbally assaulted me and always made allusion to my illness and that nobody would accept me with HIV if I left him. I realized that the relationship was unhealthy and over. One day, I gathered courage and left him because I no longer felt safe living with him (Participant 8, first interview)

#### Rejection and abandonment

A participant commented on rejection and abandonment by her intimate partner after HIV disclosure:

I was rejected. He rejected me. I had disclosed my HIV positive status to him when we met and he told me he had no problem with it but after a few months he left me.(Participant 5, first interview)

Another participant who disclosed to her husband said:

He knew I was infected through rape during the war but he left me for another woman because he could not digest the fact that I became HIV positive. He abandoned me and my children. (Participant 4, first interview)

#### Gossips

Most of the women reported that they liked participating in socio-cultural activities evident in the African tradition where it is the norm to belong to a community or small groupings. But they experienced gossips from the community, as one woman commented:

When we meet at the clinic (AIDS clinic) “juju house” (nickname for clinic), nobody greets or talks to the others. We pretend not to recognize anyone but back in town people will know who attended the clinic. (Participant 9, first interview)

Similarly another woman said:

Someone told me that it was written in a newspaper that my husband left me because of my HIV. I searched and got a copy of that newspaper, but could not find any mention of my divorce on it. I don’t know who told them. I believe it is through gossip that they knew of my HIV positive status. (Participant 10, first interview)

A participant explained that in her African community, HIV/AIDS is coded in their dialect and simply referred to as the *“4 lettered word”*, meaning AIDS, during conversations involving someone living with HIV/AIDS.

## Discussion

The main salient result is that all HIV positive SSA women in our study actively hid their diagnosis to a greater or lesser extent from others, sometimes including their intimate partners, children and caregivers. As is the case in previous studies [55–57], our research findings present a mixed picture with no simple answer for HIV disclosure. Disclosure is a complex choice, which is often difficult and a life-long process. It can be dangerous, especially if power imbalances in relationships favor men. We found out that most participants in balanced relationships did not regret disclosing their HIV positive status. Stigma and discrimination were found to be major impeding factors for disclosure. One particular form of stigma that emerged from the interviews was self-stigma. Self-stigma among SSA women encompasses denial, secrecy, silence, shame and avoidance.

Another reason for non-disclosure was fear of disrupting relationships, violence, rejection and abandonment, and these were actually experienced by a substantial proportion of the participants. Keeping their HIV positive status secret was paramount in their effort to cope with their illness and resulted in sometimes drastic coping strategies. Disclosure was best managed by being selective in revealing the illness (only to “relevant others”), and by decision making.

A limitation of this study is that a high number of SSA women with HIV/AIDS who were invited for this study refused to be interviewed because they feared that their HIV positive status would be revealed by participating, possibly leading to some selection bias. Our short inquiry with the non-participants did reveal that most of them refused to participate because the researcher herself is of African origin and the high stigma associated to HIV/AIDS disease in this culture. Most of the participants manifestly claimed that they would have accepted being interviewed if the researcher had not been of African origin, highlighting the importance of context and culture on HIV disclosure. Another limitation of the study is that women who are ‘self-identifying’ in public might have different responses to the issue of disclosure than women who were recruited by healthcare professionals. However, their disclosure was also limited to other participants at the conference who were also HIV-positive.

The strength of our study is therefore the combination of different methods, including interviews with patients and their caregivers and observations. This kind of triangulation seemed to be highly suitable for exploring disclosure intent among these HIV positive SSA migrant women, their reasons to disclose or not to disclose, and their way of dealing with their illness and disclosure or non-disclosure. It also highlights the importance of qualitative research, suitable for revealing deep-rooted fears among SSA migrant women of being labeled as HIV positive.

Our findings show that avoiding disclosure by keeping their status secret made the HIV positive SSA women feel resilient, with some sense of control over their lives, which they claim has become chaotic because of the HIV infection. In not disclosing their status and with no visible signs of HIV, they felt able to maintain their self-esteem and still benefit from socio-cultural networking. SSA migrant women with HIV/AIDS in Belgium, unlike most of their counterparts in Africa, have no obligation to disclose their HIV status because they need no financial or social support from families and friends. The cost of treatment, care and medication is mainly covered by national health insurance contributions, which is not the case in most SSA countries where families and friends pay for these services, bestowing on them the right to know the health condition they are requested or obliged to pay for.

Our findings refute the assumption that disclosure of HIV status is easier for SSA migrant women living in Belgium, with easy access combined antiretroviral therapy (cART) [58]. This study illustrates that the behavior and attitudes of SSA migrant women in relation to disclosure of HIV/AIDS status have not really changed despite the fact that they have migrated away from SSA.[59] As HIV in this group of women is largely transmitted through heterosexual contact, understanding gender, sexuality and HIV/AIDS linkage is important. Gender norms prescribing male dominance over women in the African communities make women more vulnerable to HIV prior to migration and in their new country of residence. Most often, female partners ignore male partners’ risky sexual behaviors for socio-cultural, economic and emotional reasons; unlike for men, concurrent sexual partnerships are not tolerated among women [60,61].

The support and empathy from intimate partners, families and friends received by most participants after disclosure of their positive status contributed and improved their general well-being and life with HIV/AIDS. Consequently, these women could better adhere to therapy after sharing positive diagnosis with their relevant others. Disclosing to an intimate partner who shares the worries and concerns related to the HIV disease strengthens the desire of the women to maintain a healthy lifestyle. They are better able to prevent the progression of the disease. The burden of living with the disease does not only lie on their shoulders alone after disclosure. Their ‘relevant others’ help to restore their integrity and self-perception through support and empathy.

Trauma was the immediate consequence of the HIV positive status diagnosis according to this study and these women had to learn to incorporate new identities into their lives as women, mothers and spouses within societies that stigmatize because of diseases. The women are aware of the stigma attached to being HIV positive and the fear of stigma is present even when not actually experienced. Fear, shame and blame remain similar factors that prevent SSA migrant women with HIV/AIDS from disclosing their positive status. The study revealed that most of the participants use avoidant coping strategies to deal and live with their HIV/AIDS diagnosis, trauma and stress. This is in line with other studies that have also identified stigma, in all its facets, as a major factor impeding the disclosure of HIV/AIDS status [62–66].

It is well documented that many people would like to keep their health situation confidential and there are some illnesses where the degree of confidentiality is higher than in others [67–69]. Participants were afraid of the fact that by participating in the study, the African diaspora in Belgium and subsequently in Africa would become aware of their HIV positive status. The ever- present mental suffering and pain in hiding their status from relatives and even intimate partners and children was evident in the study. However, it can be argued that women recruited through snowball sampling at HIV conferences seemed to be more open since they had at least disclosed to a “public community” and other women present at the HIV conference. However, in these groups non-HIV positive persons are not allowed to be present and members of the group are required to respect confidentiality.

Previous studies have shown that disclosing only to health care providers or another HIV positive person who lives with the virus is never the same as disclosing to close family members and friends who may have common daily chores, thus spending more time together with the HIV/AIDS infected woman [70]. It is evidenced in the literature that the HIV/AIDS pandemic is a shared responsibility between and among all infected and affected by the disease[71] but in this study, most HIV/AIDS infected women did not believe in sharing this responsibility because they experienced, anticipated or perceived negative disclosure outcomes. To a majority of these women, HIV/AIDS does not exempt them from their caregiving roles expected by family members and the society. Thus family and other social dynamics should not be disrupted by HIV/AIDS.

All the participants acknowledged the positive role of HIV care professionals in their constant fight to reconstruct and restructure their lives after the HIV diagnosis. It was interesting to find out that the women selected health care experts to whom they disclosed their status. For example, to most of the women disclosure to a general practitioner (GP) was found to be unnecessary because they could directly appeal to their treating physicians and nurses at the ARCs whenever they had any health issues that needed to be taken care of. Dentists and other health professionals not directly concerned with the treatment and care of HIV/AIDS were often not informed of their HIV status. Pharmacists in their neighborhoods were avoided, because they did not want to be identified as HIV/AIDS patients by people close to their homes.

“Living in denial” by refusing to accept HIV positive status and also refusing to disclose are strategies used by some participants to cope with their illness. Studies have shown that talking openly about HIV positive status serves as a means of adapting daily life to the medical condition [72] but this was not the case with most participants in this study. Disclosing their HIV/AIDS status for the purpose of this study could be termed ‘professional-assisted disclosure’ [73] whereby the disclosure to other people came as a result of invitations from health professionals to participate in this study. The power derived from non-disclosure is employed with legitimacy because they have a better understanding about living with HIV/AIDS. Thus, to these women non-disclosure of HIV status is “keeping their secret”, a strategy they want to cling to as long as they can. The disclosure privilege is recognized by several countries, including Belgium, where communities have created special provisions for the protection of privacy of HIV patients.

The socio-cultural background of the participants played a major role in their decision not to disclose, as seen in similar studies[74], because of the fear of stigma and discrimination, breach of confidentiality and distrust of other SSA migrants. Social dynamics influence HIV disclosure and despite the volume of this evidence and the contribution it can make towards a better understanding of factors influencing HIV status disclosure, research is still lacking. Understanding the different reasons for non-disclosure of HIV status by SSA migrant women living with HIV/AIDS could help in the development of sustainable interventions that could guide actions to support these women in their unique realities and also help them to transform and reconstruct their broken sense of social value [75].

Concerns about privacy and confidentiality may hinder patients from receiving high-quality collaborative treatment and care and thwart HIV/AIDS prevention efforts in an era when new technologies have transformed HIV/AIDS into a clinically treatable disease [76]. The reasons to disclose or not can have advantages and disadvantages to both HIV-positive women and their ‘relevant others’. The women who did not disclose or delayed HIV status disclosure to their children intended to protect their children from stigma and discrimination and the anxiety of having a mother with a life-threatening disease. However, there were no claims from the study that the children who knew of their mothers’ positive status experienced stigma and discrimination. Conversely, those women who disclosed to their children did not experience blame nor was their ‘secret’ revealed. Rather, these children became confidants and supported their mothers in their daily HIV/AIDS treatment and care. What is important in disclosing status is that the potential benefits of the disclosure should outweigh the potential losses. Fear of disclosure may lead to lost opportunities for the prevention of new infections and the ability to access appropriate treatment, care and support. The extent to which SSA migrant women with HIV disclose their infection depends on the existence of “choice freedom or choice disability”, which may have important implications in the transmission and prevention of HIV amongst their communities.

Previous studies have shown that choice disability (disparity in educational level, poverty, experienced sexual violence, intimate partner violence, partner income disparity or multiple partners) may be a driver of HIV/AIDS and may also influence HIV prevention decisions [77]. Our study reiterated some choice-disablement mechanisms among those SSA migrant women who chose not to disclose their HIV positive status. However, those who had freedom of choice did exercise this freedom through selective disclosure. Therefore, it is important to understand the choice-disablement that may affect the ability of SSA migrant women to make choices in their lives with HIV/AIDS. They should be encouraged by HIV experts to disclose to their intimate partners when they are ready to handle disclosure outcomes. Women cannot choose to share a responsibility if others will not share that responsibility. Therefore, it is imperative to address disclosure outcomes by educating the community on the need to be a partner in the fight to reduce HIV disclosure negative outcomes and focus on the benefits of disclosure at all levels.

When the community is involved to prevent onward HIV transmission through interventions that meet ethical imperatives, respect the dignity and autonomy of infected and affected individuals, and encourage voluntary testing, people with HIV/AIDS will cease to bear the sole burden of preventing HIV transmission. Research should focus on interventions that reduce choice—disablement through concerted efforts and education of the community in favor of SSA migrant women and people affected by choice disability.

## Conclusions

The HIV positive status disclosure is vital for health promotion, social support, wellbeing and the prevention of the disease.[42] However, our findings point out that HIV disclosure is a personal and complex undertaking and usually not a one-step process. Our findings also illustrate that there is a tension between a call for disclosure as a prevention measure and the understanding of women as potentially threatened by such disclosure, physically and socially. Therefore caregivers should be aware of both the benefits and the potential risks of disclosing the positive status. Women need to be prepared and supported during the whole process of disclosure. Building a support network to protect against potential ‘risky’ disclosure might be a useful strategy. This process might be facilitated by greater openness in the community as a way to reduce negative responses to status disclosure. The caregivers’ role is vital and therefore HIV disclosure should be systematically incorporated into HIV post-test counseling and primary care services. Culturally-aware providers should support women by providing information on specific governmental and non-governmental institutions to contact for assistance in case of problems related to disclosure, such as intimate partner violence, abandonment, humiliation and rejection. We recommend that interventions should not only focus on individual self-disclosure but should be more community-oriented as well, through the active involvement and participation of the community in HIV/AIDS education.
